# Baseline Blood CD8^+^ T Cell Activation Potency Discriminates Responders from Non-Responders to Immune Checkpoint Inhibition Combined with Stereotactic Radiotherapy in Non-Small-Cell Lung Cancer

**DOI:** 10.3390/cancers16142592

**Published:** 2024-07-19

**Authors:** Hanneke Kievit, M. Benthe Muntinghe-Wagenaar, Wayel H. Abdulahad, Abraham Rutgers, Lucie B. M. Hijmering-Kappelle, Birgitta I. Hiddinga, J. Fred Ubbels, Robin Wijsman, Marcel J. van der Leij, Johan Bijzet, Harry J. M. Groen, Huib A. M. Kerstjens, Anthonie J. van der Wekken, Bart-Jan Kroesen, T. Jeroen N. Hiltermann

**Affiliations:** 1 Department of Pulmonology, University of Groningen, University Medical Center Groningen, 9713 GZ Groningen, The Netherlandsh.a.m.kerstjens@umcg.nl (H.A.M.K.);; 2 Department of Rheumatology and Clinical Immunology, University of Groningen, University Medical Center Groningen, 9713 GZ Groningen, The Netherlands; 3 Department of Radiation Oncology, University of Groningen, University Medical Center Groningen, 9713 GZ Groningen, The Netherlands; 4 Department of Laboratory Medicine, Medical Immunology Laboratory, University of Groningen, University Medical Center Groningen, 9713 GZ Groningen, The Netherlands

**Keywords:** NSCLC, immune checkpoint inhibition, immunotherapy, biomarker, T cell function assay, cytokines, liquid biopsy

## Abstract

**Simple Summary:**

Cancer may be recognized by the immune system. For patients with non-small-cell lung cancer (NSCLC), immune checkpoint inhibitors (ICI) are a first-line treatment in most patients. However, most of these patients do not respond to ICI, implying that the treatment is ineffective, where it may have relevant side effects. Currently, there is no solid biomarker to predict response to ICI. Thus, there is an urgent need for a new biomarker to predict this response, preferably via minimally invasive techniques. We tested the potency of T cells to be activated ex vivo in the peripheral blood of patients with advanced NSCLC. We found an increased ability to activate CD8^+^ T cells and produce intracellular IL-2 in peripheral CD8^+^ T cells in patients that respond to ICI compared to non-responders and healthy controls before the start of ICI. The potency of peripheral T cells to be activated before treatment seems a promising biomarker.

**Abstract:**

Background: Tumor-infiltrating immune cells have been correlated with prognosis for patients treated with immune checkpoint inhibitor (ICI) treatment of various cancers. However, no robust biomarker has been described to predict treatment response yet. We hypothesized that the activation potency of circulating T cells may predict response to ICI treatment. Methods: An exploratory analysis was conducted to investigate the association between the response to immune checkpoint inhibition (ICI) combined with stereotactic radiotherapy (SBRT) and the potency of circulating T cells to be activated. Blood-derived lymphocytes from 14 patients were stimulated ex vivo with, among others, Staphylococcal enterotoxin B (SEB) and compared to healthy controls (HCs). Patients were grouped into responders (>median progression free survival (PFS)) and non-responders (<median PFS). The expression of the T cell activation marker CD69 and intracellular cytokines (IL-2, IFNγ, TNFα) in both CD4^+^ and CD8^+^ T cells in response to stimulation was measured using flow cytometry. In addition, serum levels of BAFF, IFNγ, and IL-2 receptor (sIL-2R) were measured by Luminex. Results: At baseline, a higher percentage of activated CD8^+^ T cells (15.8% vs. 3.5% (*p* = <0.01)) and IL-2^+^CD69^+^CD8^+^ T cells (8.8% vs. 2.9% (*p* = 0.02)) was observed in responders compared to non-responders upon ex vivo stimulation with SEB. The concurrently measured serum cytokine levels were not different between responders and non-responders. Conclusion: Baseline blood CD8^+^ T cell activation potency, measured by intracellular cytokine production after ex vivo stimulation, is a potential biomarker to discriminate responders from non-responders to SBRT combined with ICI.

## 1. Introduction

Cancers can be recognized by the immune system, and immune checkpoint inhibition (ICI) presents an important treatment option, particularly for Non-Small-Cell Lung Cancer (NSCLC) [1,2,3,4]. However, not all tumors exhibit a favorable outcome to ICI. “Hot” tumors, characterized by a heightened T cell inflammatory profile, demonstrate a markedly superior response to ICI compared to “cold” tumors, which are defined by a low or absent T cell inflammatory profile. Resected tumors from individuals who respond to neoadjuvant ICI show a dense presence of immune activation, including infiltrating lymphocytes, macrophages, and tertiary lymphoid structures (TLS) [5,6,7]. The abundance of tumor-infiltrating CD8^+^ T cells in both the TLS and the tumor microenvironment has been correlated with a more favorable prognosis in various cancers, including lung cancer [8,9,10].

To this date, the only biomarker in clinical practice used to predict the responsiveness of NSCLC to ICI is the expression of PD-L1 on tumor cells. In advanced lung cancer, tissue biopsy is essential for diagnosis and treatment decisions [11,12]. However, tissue availability is usually limited owing to small biopsies or cytological specimens. Consequently, there is ongoing exploration of less invasive techniques to obtain biomarkers, particularly those from peripheral blood sampling, to predict tumor response. In patients with NSCLC who received second-line nivolumab, a pre-treatment neutrophil-to-lymphocyte ratio (NLR) below 5 was associated with an extended progression free survival (PFS) and overall survival (OS) compared to a higher NLR (≥5) [13,14]. Furthermore, elevated baseline levels of serum Interleukin 6 (IL-6) levels were associated with a shortened PFS and OS [15]. Flow cytometric analysis of peripheral blood lymphocytes of NSCLC patients receiving ICI treatment revealed that response to treatment was associated with a higher percentage of CD62lowCD4^+^ T cells, whereas non-responders were characterized by a higher percentage of CD25^+^FOXP3^+^CD4^+^ T cells prior to treatment [16]. Additionally, higher levels of CD4^+^ T cells, CD4^+^/CD8^+^cell ratios, and absolute numbers of natural killer cells (NK cells) were associated with response to ICI [17].

Beyond the conventional assessment of immune status through flow cytometry, various aspects of T cell function, such as cytokine production following mitogenic ex vivo stimulation of T cells, can be assessed. Cytokines, such as tumor necrosis factor alpha (TNFα), interferon gamma (IFNγ), and interleukin 2 (IL-2) play an important role in modulating the immune response. Both IFNγ and IL-2 are known to promote the cytotoxicity of CD8^+^ cells and NK cells and to stimulate the differentiation of CD4^+^ T cells into Th1 helper cells [10,18]. In patients with NSCLC, the IFNγ signature is associated with prolonged survival [19].

We hypothesized that peripheral blood lymphocytes from NSCLC patients at baseline who respond to ICI exhibit increased sensitivity to mitogenic stimulation, leading to an augmented production of intracellular cytokines. If substantiated, this could potentially serve as a viable biomarker for selecting patients for ICI treatment. In this study we evaluated the baseline expression of the T cell activation marker CD69 and intracellular cytokines (IL-2, IFNγ, TNFα) in both CD4^+^ and CD8^+^ T cells after ex vivo stimulation and their association with response to combined ICI and SBRT treatment.

## 2. Materials and Methods

This exploratory analysis was part of a phase 1 study on safety and tolerability in patients with stage IIIB/IV NSCLC treated with stereotactic radiotherapy (SBRT) on a part of the primary tumor, combined with ICI treatment. ICI was administered in three different regimes in sequential cohorts as ≥2nd line treatment after platinum-based chemotherapy. The 1st cohort (*n* = 3) received durvalumab (PD-L1 inhibitor), while the 2nd and 3rd cohorts (both *n* = 6) received a combination of durvalumab and tremelimumab (CTLA-4 inhibitor) followed by durvalumab monotherapy. All patients were irradiated on the primary tumor (1 × 20 Gy on 9 cc) one week after the 1st dose of ICI. For further details regarding inclusion and exclusion criteria, patient characteristics, and full trial design of this phase 1 study, the SICI (stereotactic radiotherapy and immune checkpoint inhibition) trial, we refer to Kievit et al. [20]. Patients were grouped into responders (>median progression free survival (PFS)) and non-responders (≤median PFS).

Exploratory endpoints in this study were the correlation of response and survival with lymphocyte count, T cell activation potency, and the correlation between intracellular and serum cytokines at baseline just before the start of the combined ICI and SBRT treatment. PFS was defined from date of start of the treatment to the date of the first documented date of progression or death by any cause. OS was defined from date of start of the treatment to date of death. If a patient had not died, the OS was censored at the date of last follow-up. Cutoff date was 1 July 2024.

### 2.1. Lymphocyte Count 

Blood lymphocyte counts were differentiated into CD45^+^CD3^+^ T cells, CD45^+^CD3^+^CD4^+^ T helper cells, CD45^+^CD3^+^CD8^+^ cytotoxic T cells, CD45^+^CD19^+^ B cells and CD45^+^CD16^+^CD56^+^ NK cells. The NLR was calculated by dividing the absolute neutrophil count by absolute lymphocyte count and marked as low (<5) or high (≥5). 

### 2.2. T Cell Activation Analysis 

Potency of T cell activation was assessed using flow cytometric analysis of peripheral blood T cellT cells and compared to simultaneously included healthy controls, a standard assay that is used for the diagnosis of immune deficiencies as described by Stam et al. [21]. In short, whole blood was activated ex vivo using one of the following mitogens or antigens: 5 ug/mL Staphylococcal enterotoxin B (SEB; Sigma, Deisenhofen, Germany), anti-CD3 (aCD3; 10% *v*/*v* WT32 hybridoma culture supernatant), 5 ug/mL Phyto haemaglutine (PHA; Remel, Lenexa, KS, USA), a cocktail of 15 Lf/mL tetanus toxoid and diphtheria toxoid (cocktail; Netherlands Vaccine Institute; NVI, Bilthoven, The Netherlands), and 0.5 TE/mL purified protein derivative Tuberculosis (PPD; NVI, Bilthoven, The Netherlands). To block cytokine release from cells, 10 µg/mL brefeldin A (BFA; Sigma-Aldrich, St. Louis, MI, USA) was added to each sample. Next, samples were incubated for 24 h at 37 °C with 5% CO_2_. Post-incubation, samples were treated with 10 µL of 40 mM EDTA in PBS for 10 min to inhibit activated cell adhesion. Following this, red blood cells were lysed, and white blood cells were fixed by adding 2 mL of FACS lysing solution (Becton Dickinson, Franklin Lakes, NJ, USA) for 10 min at room temperature, followed by a PBS wash with 1% bovine serum albumin (BSA). Next, each sample was permeabilized with 500 µL of Perm II (Becton Dickinson) containing varying concentrations and/or combinations of Pacific Blue (PB) and/or Pacific Orange (PO) dyes (Invitrogen, Carlsbad, CA, USA) to facilitate fluorescent cell barcoding (FCB) as depicted in Figure S1. Unstimulated samples were stained with 1.25 µg PB and 10 µg PO, while samples stimulated with PPD, SEB, aCD3 (WT32), cocktail, and PHA were stained with 0 µg PB and 0 µg PO, 1,25 µg PB and 0 µg PO, 10 µg PB and 0 µg PO, 0 µg PB and 10 µg PO, and 10 µg PB and 10 µg PO, respectively. After 10 min of incubation at room temperature in the dark, samples were washed and resuspended in PBS with 20% fetal calf serum (FCS). T cell activation potency was assessed by evaluating the expression of CD69 and intracellular cytokines (IL-2, IFNγ, TNFα). Alongside each patient’s sample, we included a healthy control sample in our test. Flow cytometric analysis was done on 50,000 recorded events per analysis using a FACSCanto-II flow cytometer (Beckton Dickinson Biosciences, San Jose, CA, USA). Diva v9.3.1 (Beckton Dickinson) and Kaluza v2.2 (Beckman Coulter, Brea, CA, USA) software was used for gating and flow cytometry data analysis. The gating strategy is shown in Figure S1. Patients were divided into above median PFS (responders; 0 R) and below median PFS (non-responders; NR). We compared intracellular cytokine production in both CD4^+^ and CD8^+^ T cells between both groups and with healthy controls (HC) using the Mann–Whitney U test.

### 2.3. Serum Cytokines 

The intracellular cytokine production of IL-2, IFNγ and TNFα was correlated to serum concentrations of BAFF/BLyS/TNFSF13B, CD25/IL-2R alpha and IFNγ measured by multiplex Luminex as described by the protocol of the manufacturer (R&D, Austin, TX, USA). For an overall indication of T cell and B cell activation, the Luminex panel was expanded with CCL17/TARC, IL-6 and CXCL13/BLC/BCA-1.

### 2.4. Statistics 

Relationships between lymphocyte count and response, as well as the relationship between serum cytokines and response were analyzed with the Mann–Whitney U test. The same test was used to compare differences in T cell activation assay between responders, non-responders, and healthy controls. Spearman’s correlations were calculated between survival and NLR and the lymphocyte count and to assess relations between intracellular and serum cytokines. 

### 2.5. Ethical and Regulatory Requirements 

This study was performed in accordance with ethical principles that have their origin in the Declaration of Helsinki and are consistent with ICH-Good Clinical Practice, and applicable regulatory requirements concerning subject data protection. The study protocol was approved by the local medical ethic committee (ICTRP: NL-OMON44296; EudraCT: 2017–002797-39). All patients gave their written informed consent prior to the start of any study related procedures.

## 3. Results

Fifteen patients were included in the phase 1 SICI trial. In one patient, blood storage failed due to technical issues. Therefore, the data of 14 patients (8 non-responders and 6 responders) were available for the exploratory analysis at baseline. Patients categorized as responder by the mean PFS had either stable disease or partial response as best response to treatment and all non-responders had progressive disease as best response. As an internal control of the T cell activation assay, 14 healthy controls served as reference. Healthy controls were younger compared to the NSCLC patients (median age 48 years vs. 64 years, *p* < 0.001) and were more often female (71% vs. 14%, *p* = 0.002). None of the patients used corticosteroids at the start of treatment. Overall survival (OS) was 12 months (range 1—not reached) and superior for responders compared to non-responders (Figure S2 and phase 1 SICI trial [20]).

### 3.1. Lymphocyte Count 

**Figure 1:**
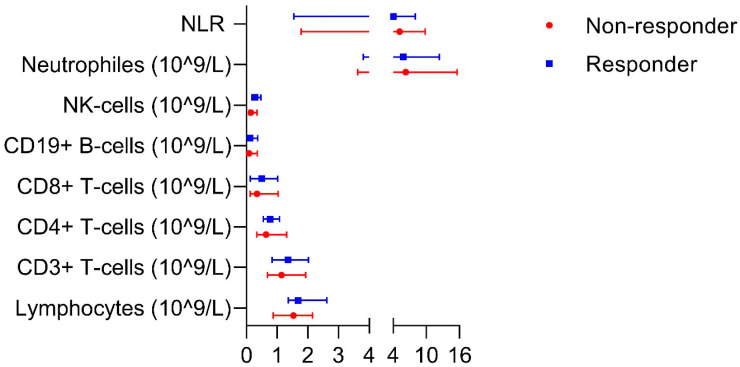
Blood lymphocytes between responders and non-responders at baseline. Data are presented as median with its range. No significant differences were observed, *p* > 0.14. NK cells: natural killer cells. NLR: neutrophile-to-lymphocyte ratio.

There was no significant difference in blood lymphocytes at baseline between responders and non-responders (Figure 1, Table S1). For all patients, OS showed a significant negative correlation with the neutrophil-to-lymphocyte ratio NLR (spearman’s rho -0.55, *p* = 0.04). Furthermore, OS was positively correlated with all lymphocytes (0.71, *p* = 0.005), CD3^+^ T cells (0.73, *p* = 0.003) and CD8^+^ T cells (0.63, *p* = 0.016) (Table S2). All other correlations between the lymphocyte count and PFS or OS were not significantly different.

### 3.2. T Cell Activation Assay at Baseline

**Figure 2:**
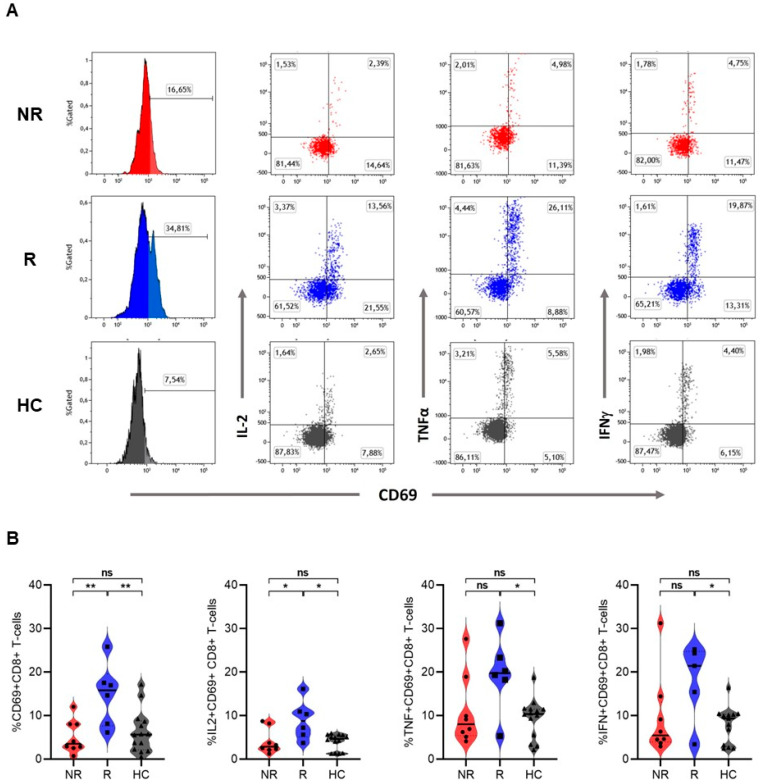
Activation of intracellular cytokine expression in CD8^+^ T cells after stimulation with SEB. (**A**) Representative flow cytometry plots of SEB-stimulated CD8^+^ T cells showing CD69 expression alone (histograms) or CD69 expression in combination with expression of intracellular cytokines IL-2, TNFα, IFNγ (dot-plots) in a non-responder (NR; upper plots), a responder (R; middle plots), and an age- and sex-matched healthy control (HC; lower plots). Values in each gate represent the percentage of positive cells; (**B**) frequencies of CD69, IL-2, TNFα, and IFNγ expression within responding CD8^+^ T cells from NR (*n* = 8), R (*n* = 6) and HC (*n* = 14) after in vitro stimulation with SEB. Horizontal lines represent the median percentage. *p*-values were calculated using the Mann–Whitney U test. *: *p* <0.05. **: *p* <0.001. ns: non-significant.

Before the start of treatment, responders had significantly higher percentages of activated (CD69^+^)CD8^+^ T cells, especially those producing IL-2 in response to SEB stimulation, compared to both non-responders (CD69^+^CD8^+^ 15.8% vs. 3.5% (*p* = 0.008); IL-2^+^CD69^+^CD8^+^ 8.8% vs. 2.9% (*p* = 0.02)) and healthy controls (5.6% (*p* = 0.009) and 4.7%, respectively (*p* = 0.001)). No difference in the expression of IL-2 was observed between T cells of non-responders and healthy controls. In addition, significantly higher expression of TNFα and IFNγ was observed in activated CD8^+^ T cells of responders when compared to healthy controls (resp 19.8% vs. 10.4%; *p* = 0.01 and 21.4% vs. 9.3%; *p* = 0.03). The expression of TNFα and IFNγ by T cells was not different between responders and non-responders (TNFα and IFNγ of non-responders resp. 8.0% and 5.5%) (Figure 2). No difference was observed in the percentage of activated CD8^+^ T cells after stimulation with anti-CD3 between responders, non-responders, and healthy controls. Stimulation with the strong mitogen PHA resulted in strong T cell activation, evident from significant CD69 upregulation without observable differences between responders, non-responders, and healthy controls in activated T cells and intracellular cytokine production. Stimulation with the weak antigenic cocktail or PPD resulted in almost no stimulation of both CD8^+^ and CD4^+^ T cells with no differences between groups (Table S3). 

**Figure 3:**
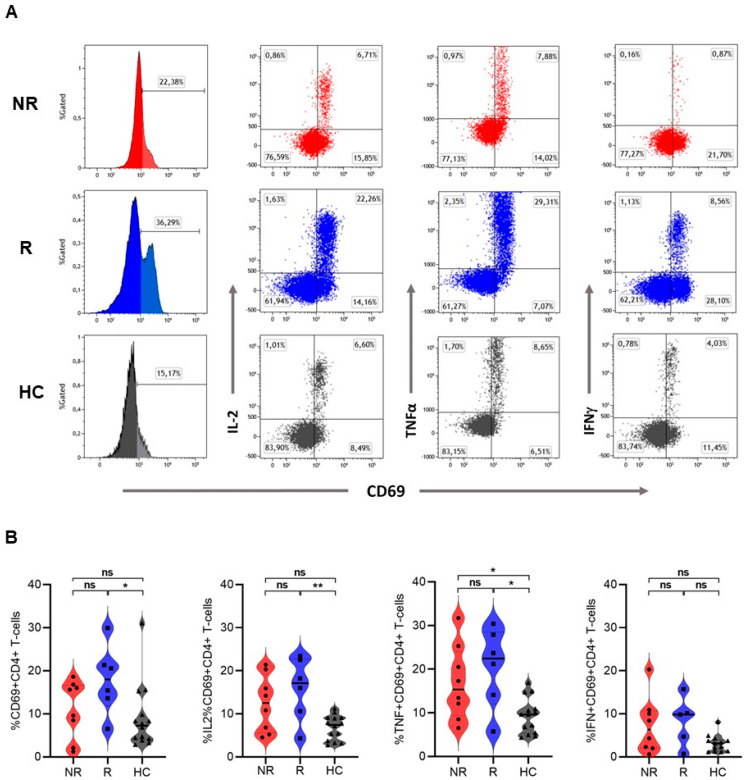
Activation and intracellular cytokine expression in CD4^+^ T cells after stimulation with SEB. (**A**) Representative flow cytometry plots from SEB-stimulated CD4^+^ T cells showing CD69 expression alone (histograms) or in CD69 expression combination with expression of intracellular cytokines IL-2, TNFα, and IFNγ (dot-plots) in a non-responder (NR; upper plots), a responder (R; middle plots), and an age- and sex-matched healthy control (HC; lower plots). Values in each gate represent the percentage of positive cells. (**B**) Frequencies of CD69, IL-2, TNFα, and IFNγ expression within responding CD4^+^ T cells from NR (*n* = 8), R (*n* = 6), and HC (*n* = 14) after in vitro stimulation with SEB. Horizontal lines represent the median percentage. *p*-values were calculated using the Mann–Whitney U test. *: *p* <0.05. **: *p* <0.001. ns: non-significant.

In contrast to CD8^+^ T cells, in activated (CD69^+^) CD4^+^ T cells, no significant differences were observed between responders and non-responders after stimulation with SEB (CD69^+^CD4^+^ 18% vs. 12.6% (*p* = 0.23), IL-2^+^CD69^+^CD4^+^ 17.1% vs. 12.5% (*p* = 0.41), TNFα^+^CD69^+^CD4^+^ 22.5% vs. 15.4% (*p* = 0.49) and IFNγ^+^CD69^+^CD4^+^ resp 9.8% and 6.3% (*p* = 0.10). Compared to healthy controls, only responders had significant higher percentages of activated (CD69^+^) CD4^+^ T cells when stimulated with SEB, including higher expression of IL-2 and TNFα (CD69^+^CD4^+^ 18% vs. 7.3% (*p* = 0.03), IL-2^+^CD69^+^CD4^+^ 17.1% vs. 7.4% (*p* = 0.009), and TNFα^+^CD69^+^CD4^+^ 22.5% vs. 9.6% (*p* = 0.02)). No significant difference was observed in IFNγ expression between responders and healthy controls (IFNγ^+^CD69^+^CD4^+^ 9.8% vs. 3.1% (*p* = 0.72)). TNFα expression only, was significantly different between non-responders and healthy controls (TNFα^+^CD69^+^CD4^+^15.4% vs. 9.4% (*p* = 0.03), Figure 3). For CD4^+^ T cells stimulated with anti-CD3, a similar cytokine response as with SEB was observed (Table S3).

### 3.3. Luminex Assay

**Figure 4:**
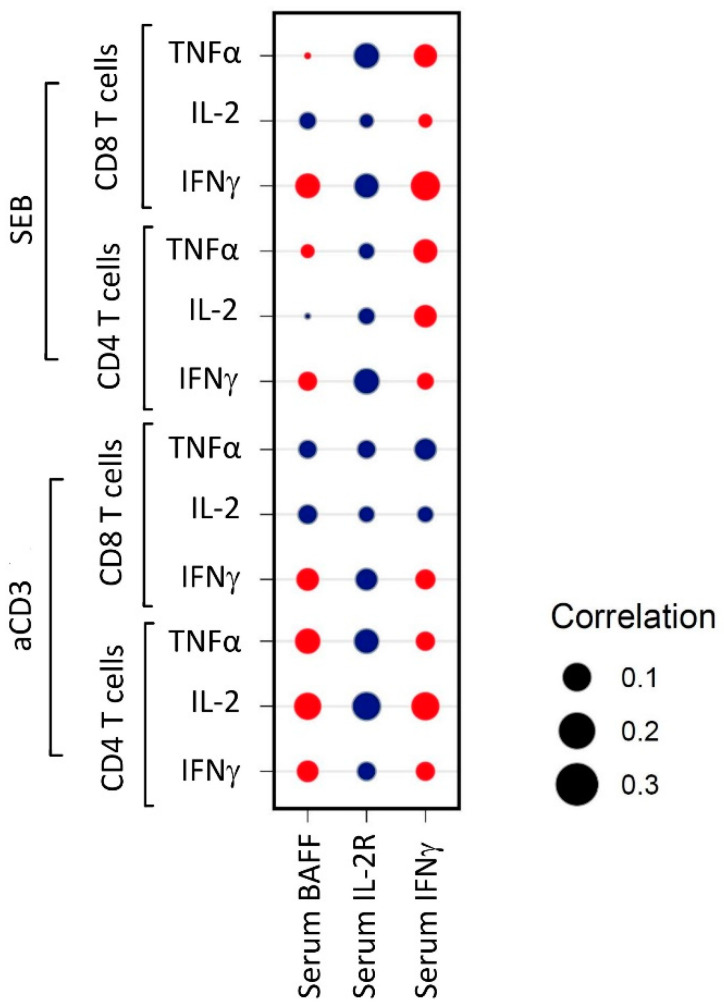
Correlation between baseline intracellular and serum cytokine expression after stimulation with SEB and anti-CD3. A red circle indicates a positive correlation, a blue circle a negative correlation. aCD3: anti CD3. BAFF: B-cell-activating factor belonging to the TNF family. IFNγ: interferon gamma. IL-2: interleukin 2. SEB: Staphylococcal enterotoxin B. TNFα: tumor necrosis factor alpha.

At baseline, serum cytokines (IFNγ, IL-2 (CD25/IL-2R alpha), TNFα (BAFF/BLyS/TNFSF13B), CCL17 (CCL17/TARC), IL-6, and CXCL13 (CXCL13/BLC/BCA-1)), which were selected based on complementarity, appearance, and function in immune activation, were not different between responders and non-responders (Table S4). In addition, there was no correlation between intracellular IL-2, IFNγ and TNFα expression by T cells and serum levels of three selected cytokines (BAFF/BLyS/TNFSF13B, CD25/IL-2R alpha, IFNγ) (Figure 4).

## 4. Discussion

In this study, we provide evidence that the potency of T cell activation at baseline, as assessed through stimulated intracellular cytokine production, may serve as a non-invasive biomarker to discriminate responders from non-responders to ICI. Specifically, our findings indicate increased expression of cytokines, particularly IL-2 expression by CD8^+^ T cells following ex vivo stimulation at baseline in patients who turned out to respond to ICI (in combination with SBRT to the primary tumor). 

To the best of our knowledge, this is the first study in NSCLC exploring peripheral T cell activation potency as a predictor of response to ICI. T cell activation was assessed by measurement of intracellular cytokines (IL-2, IFNγ, TNFα) following stimulation of peripheral blood T cells with PHA, a tetanus and diphtheria cocktail, PPD, anti-CD3, or SEB. Stimulation of T cells using PHA involves several cell surface receptors, most notably CD3 and CD2 and is, as such, an unselective and strong mitogenic activator of T cells that partly bypasses co-stimulation [22,23]. The strong nature of stimulation using PHA masks possible subtle differences in the activation potency of T cells between groups. In line with this, we did not observe any differences between responders, non-responders, and healthy controls in the expression of cytokines by T cells upon stimulation with PHA. In contrast, the cocktail and PPD are weak antigenic recall stimuli, only activating a subpopulation of T cells, i.e., those that were previously exposed to these stimuli. Using these weak recall stimuli, no differences were noted between the three groups analyzed here, possibly reflecting a too-weak or not-sensitive-enough readout. Using a more intermediate stimulus, such as SEB (or anti-CD3), functional differences in the activation potency of T cells derived from the different groups might be revealed. Both SEB and anti-CD3 stimulate T cells specifically via the T cell receptor (TcR)/CD3 complex, where anti-CD3 does so with higher affinity compared to SEB. As such, SEB potentially represents the most optimal mode of T cell activation to reveal intrinsic differences in the T cell activation potency between the different groups studied here. Indeed, most notable and significant differences in the responses were observed upon SEB stimulation, especially in the CD3^+^CD8^+^ T cell compartment. 

CD8^+^ T cells play an important role in the immune response to cancer and the number of CD8^+^ T cells, and their capability of infiltration into the tumor (environment) is associated with prolonged survival. Activated CD8^+^ T cells are capable of killing cancer cells, which is associated with the release of cytokines [24]. Effective activation of (cytotoxic) CD8^+^ T cells requires signaling via the TcR/CD3 complex in combination with one or more costimulatory signals. Using ICI, both anti-CTLA4 and anti-PD(L)1 enhance the activation of the T cell by blocking an inhibitory signal (the interaction between CTLA-4 and CD80 and PD-1 with PD-L1, respectively) allowing unrestrained T cell (co)stimulation [25,26]. 

In our study, the CD8^+^ T cells of responders were significantly more prone to activation at baseline compared to non-responders and healthy controls and expressed more intracellular cytokines, especially IL-2, after ex vivo stimulation with SEB. This suggests that the CD8^+^ T cells of responders have an increased potential to become activated via the TcR/CD3 complex even before starting ICI treatment than non-responders. This increased potential to be activated might reflect the observed response to ICI treatment and as such, may represent a potential baseline biomarker for ICI response. A similar difference, although non-significant, was seen in the activation potency of CD4^+^ T cells of responders and non-responders. Alternatively, the poorer responsiveness of T cells to ex vivo stimulation in non-responders may be an indication of an increased presence or activity of regulatory T cells, which has been described as a resistance mechanism to ICI treatment [16]. Alternatively, it may be an indication of exhausted CD8^+^ T cells, another resistance mechanism for immunotherapy to fail [27].

Absolute lymphocyte count, including different subtypes, was not significantly different between responders and non-responders. We compared the intracellular cytokine production of IL-2, IFNγ, and TNFα with the serum values of these cytokines. There was no correlation between the serum cytokines and T cell activation potency, nor with intracellular cytokine production upon stimulation of T cells at baseline. This is in line with the melanoma data and ICI of the research by Pedersen et al., where baseline serum derived cytokines including TNFα, IFNγ and IL-6 were not associated with PFS [28]. Cytokines released by T cells after stimulation in vivo are likely either directly used or bound by surrounding cells. Therefore, circulating serum cytokines levels may not be representative for T cell activation potency at baseline. Apparently, T cells prone to activation by ICI are thus not reflected in serum cytokines or lymphocyte subsets, and this specific characteristic needs to be measured with ex vivo stimulation in combination with intracellular cytokine expression.

Our study included patients with an indication for ≥second-line ICI. Unfortunately, we were not able to include more patients in this study. Due to advancements in knowledge, ICI became standard first-line treatment in patients with advanced NSCLC without a driver mutation during the period of our study. We realize that because of this, our sample size is limited. Nevertheless, this is an exploratory parameter and for the generalizability of our result, this should be repeated in an independent and larger cohort. We think that the use of SBRT in our patient cohort had little influence on the result because of the small treatment volumes. Radiation-induced lymphopenia is associated with multiple courses, multiple irradiated sites, and higher dose (>50 Gy) and is mainly seen when large vessels and the heart are part of the irradiated field [29,30]. Therefore, we suggest studying T cell activation in patients receiving immune checkpoint inhibition in a first-line setting. Our healthy controls were laboratory employees working on the day of blood withdrawal of the participating patients and were not age- and sex-matched with the patients. As such, we cannot rule out that differences between patients and healthy controls observed are due to differences in sex and or age between the groups included.

## 5. Conclusions

The potency to activate CD8^+^ T cells at baseline, as determined through ex vivo activation and intra-cellular cytokine production, discriminates responders from non-responders to immune checkpoint inhibition combined with stereotactic radiotherapy in our patient cohort with non-small-cell lung cancer. Further research—for instance, in first-line ICI treatment in a larger cohort—is needed to confirm this potential biomarker.

## Data Availability

The data generated in this study are available upon reasonable request from the corresponding author.

## Supplementary Materials

The following supporting information can be downloaded at: https://www.mdpi.com/article/10.3390/cancers16142592/s1, Figure S1: Representative gating strategy of quantifying cytokine-expressing CD4^+^ and CD8^+^ T cells using fluorescent cell barcoding (FCSB) technique; Figure S2: Overall survival; Table S1: Baseline characteristics; Table S2: Correlation between lymphocyte count and survival; Table S3: Median percentages of responding (CD69^+^) CD4^+^ and CD8^+^ T—cells and cytokine production after stimulation; Table S4: Median percentages of responding (CD69^+^) CD4^+^ and CD8^+^ T—cells and cytokine production after stimulation.
