# Supplementary material for: Baseline Blood CD8+ T Cell Activation Potency Discriminates Responders from Non-Responders to Immune Checkpoint Inhibition Combined with Stereotactic Radiotherapy in Non-Small-Cell Lung Cancer
Source: Cancers (Basel). 2024 Jul 19;16(14):2592. doi: 10.3390/cancers16142592 (PMC11275013; doi:10.3390/cancers16142592)
Supplement: Supplementary file 1 [file cancers-16-02592-s001.zip › cancers-3093785-supplementary.pdf]

Supplementary Figures and Tables

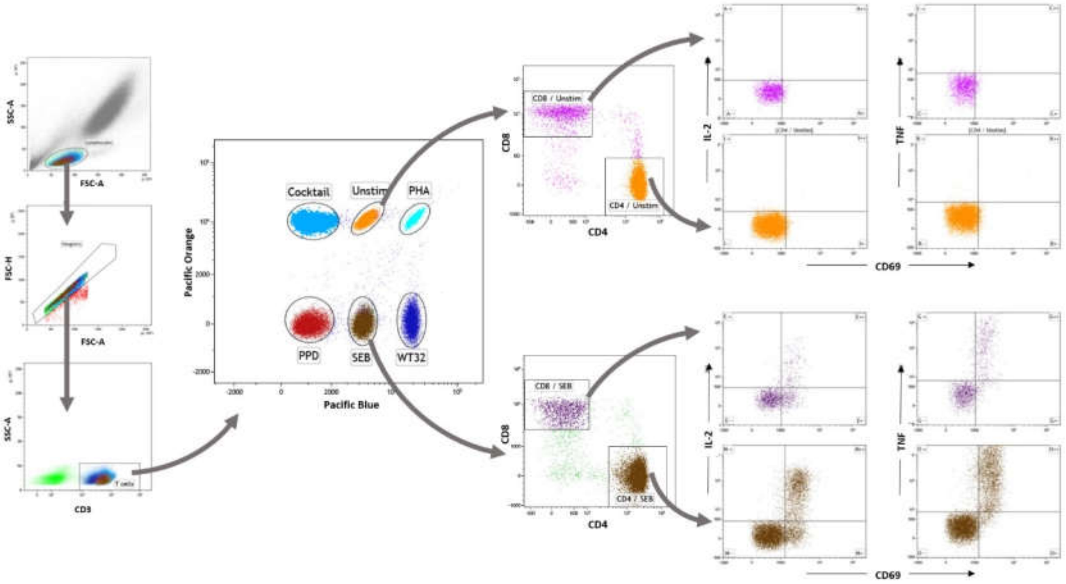

Supplementary Figure S1. Representative gating strategy of quantifying cytokine-expressing CD4⁺ and CD8⁺ T-cells using fluorescent cell barcoding (FCSB) technique. Peripheral blood lymphocytes were identified and gated based on forward (FSC) and side (SSC) scatter. After excluding doublets in the FSC-H/FSC-A plot, CD3⁺ T cells were selected and analyzed for their Pacific Blue and Pacific Orange fluorescence intensity to identify the sample origin of stimulated cells. In this example, unstimulated and SEB stimulated CD4⁺ and CD8⁺ T cells were evaluated for the expression of cytokines (IL-2 and TNF) and activation marker (CD69). Gates for the expression of cytokine and CD69 were set based on unstimulated cells and applied uniformly to stimulated cells.

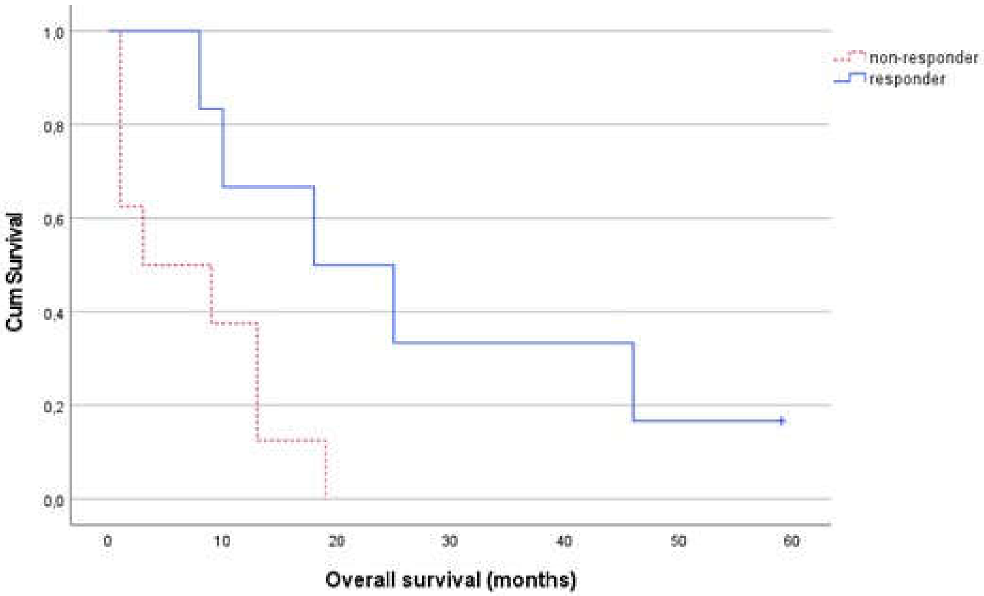

Supplementary Figure S2. Overall survival. Overall survival in months between responders (>median PFS) and non-responders (≤median PFS). Cut-off for overall survival was last date of visit before 1 July 2024.

Supplementary Table S1. Baseline characteristics

		**Non-responders (N = 8)**	**Responders (N = 6)**
Patient	Age (years)	71 [52 – 85]	63 [52 – 69]
	Sex (female)	1 (13)	1 (17)
	Tumor histology		
	AC	5 (63)	5 (84)
	SCC	3 (38)	1 (17)
	Tumor Stage		
	IVA	2 (25)	2 (33)
	IVB	6 (75)	4 (67)
	ECOG PS		
	0	2 (25)	2 (33)
	1	6 (75)	4 (67)
	Smoking status		
	Never	1 (13)	-
	Former	7 (88)	3 (50)
	Current	-	3(50)
	Alcohol usage (yes)	6 (75)	4 (67)
Lymphocyte count	Lymphocytes (10^9/L)	1.53 [0.87 – 2.16]	1.69 [1.37 – 2.62]
	CD3$^+$ T-cells (10^9/L)	1.14 [0.68 – 1.93]	1.35 [0.84 – 2.03]
	CD4+ T-cells (10^9/L)	0.64 [0.33 – 1.31]	0.77 [0.55 – 1.08]
	CD8+ T-cells (10^9/L)	0.34 [0.12 – 1.03]	0.50 [0.13 – 1.01]
	CD19+ T-cells (10^9/L)	0.08 [0.02 – 0.35]	0.12 [0.06 – 0.37]
	NK-cells (10^9/L)	0.14 [0.09 – 0.34]	0.27 [0.18 – 0.47]
	Neutrophils (10^9/L)	6.31 [3.63 – 15.58]	5.85 [3.81 – 12.38]
	NLR	5.16 [1.78 – 9.80]	4.05 [1.54 – 8.04]

Data is presented as median [range] or n (%). Non-responders had progressive disease as their best response, responders had stable disease or partial response as their best response. AC: Adenocarcinoma. SCC: Squamous cell carcinoma. ECOG PS: Eastern Cooperative Oncology Group performance score. N: number. NK-cells: Natural Killer cells. NLR: Neutrophile-to-lymphocyte ratio. No significant differences were observed p> 0.14.

Supplementary Table S2. Correlation between lymphocyte count and survival

	**Overall Survival**	**Progression Free Survival**
Lymphocytes (10^9/L)	0.71 [0.005]	0.35 [0.22]
$CD3^+$ T-cells (10^9/L)	0.73 [0.003]	0.23 [0.41]
CD4+ T-cells (10^9/L)	0.31 [0.29]	0.31 [0.27]
CD8+ T-cells (10^9/L)	0.63 [0.02]	0.14 [0.63]
CD19+ T-cells (10^9/L)	0.09 [0.76]	0.24 [0.39]
NK-cells (10^9/L)	0.21 [0.46]	0.43 [0.13]
Neutrophils (10^9/L)	-0.33 [0.25]	-0.16 [0.58]
NLR	-0.55 [0.04]	-0.29 [0.31]

Correlation is measured with Spearman's rho [P-value]. NK-cells: Natural Killer cells. NLR: Neutrophil-to-lymphocyte ratio

Supplementary Table S3. Median percentages of responding (CD69+) CD4+ and CD8+ T – cells and cytokine production after stimulation.

$CD4^+$ T – Cells (%)	Responders	Non-responders	Healthy controls
**Stimulated with SEB**			
$CD69^+$	18.0 [6.5 – 29.9]	12.6 [1.2 – 18.6]	7.3 [2.8 – 31.0]
$IL2^+CD69^+$	17.1 [4.3 – 23.4]	12.5 [4.5 – 21.4]	7.4 [2.7 – 11.2]
$IFN\gamma^+CD69^+$	9.8 [0.7 – 15.7]	6.4 [0.6 – 20.3]	3.1 [1.1 – 8.1]
$TNF\alpha^+CD69^+$	22.5 [5.7 – 30.4]	15.4 [6.5 – 31.7]	9.6 [4.3 – 16.9]
**Stimulated with aCD3**			
$CD69^+$	11.7 [1.37 – 31.0]	6.3 [0.1 – 25.9]	5.4 [0.0 – 17.6]
$IL2^+CD69^+$	1.75 [0.0 – 9.1]	0.6 [0.0 – 16.4]	0.0 [0.0 – 0.8]
$IFN\gamma^+CD69^+$	1.4 [0.0 – 5.2]	0.6 [0.0 – 19.4]	0.0 [0.0 – 1.4]
$TNF\alpha^+CD69^+$	10.3 [0.1 – 26.6]	6.2 [0.6 – 35.8]	2.5 [1.0 – 13.2]
**Stimulated with PHA**			
$CD69^+$	68.2 [33.7 – 78.2]	46.0 [17.7 – 82.4]	61.5 [12.4 – 84.4]
$IL2^+CD69^+$	29.0 [21.9 – 40.3]	21.7 [18.1 – 36.50]	29.2 [18.6 – 41.1]
$IFN\gamma^+CD69^+$	11.8 [1.9 – 27.5]	9.4 [0.9 – 67.6]	11.9 [6.0 – 30.2]
$TNF\alpha^+CD69^+$	47.0 [35.5 – 65.1]	46.4 [21.6 – 82.4]	46.5 [20.9 – 55.6]
**Stimulated with Cocktail**			
$CD69^+$	1.1 [0.5 – 2.0]	0.1 [0.0 – 1.3]	0.1 [0.0 – 2.5]
$IL2^+CD69^+$	0.1 [0.0 – 0.5]	0.1 [0.0 – 1.1]	0.0 [0.0 – 0.6]
$IFN\gamma^+CD69^+$	0.1 [0.0 – 0.2]	0.0 [0.0 – 0.3]	0.0 [0.0 – 0.5]
$TNF\alpha^+CD69^+$	0.1 [0.0 – 0.3]	0.1 [0.0 – 2.3]	0.0 [0.0 – 1.0]
**Stimulated with PPD**			
$CD69^+$	0.6 [0.0 – 3.1]	0.3 [0.0 – 2.5]	0.3 [0.0 – 1.7]
$IL2^+CD69^+$	0.0 [0.0 – 1.4]	0.2 [0.0 – 0.9]	0.0 [0.0 – 0.3]
$IFN\gamma^+CD69^+$	0.0 [0.0 – 0.3]	0.0 [0.0 – 0.5]	0.0 [0.0 – 0.4]
$TNF\alpha^+CD69^+$	0.0 [0.0 – 2.6]	0.0 [0.0 – 1.2]	0.1 [0.0 – 0.8]

$CD8^+$ T – Cells (%)	Responders	Non-responders	Healthy controls
**Stimulated with SEB**			
$CD69^+$	15.8 [6.1 – 25.8]	3.5 [0.7 – 12.0]	5.6 [0.5 – 17.1]
$IL2^+CD69^+$	8.8 [3.7 – 16.1]	2.9 [1.2 – 8.7]	4.7 [1.2 – 5.9]
$IFN\gamma^+CD69^+$	21.4 [3.4 – 25.2]	5.5 [2.9 – 31.2]	9.3 [2.4 – 16.4]
$TNF\alpha^+CD69^+$	22.5 [5.7 – 30.4]	8.0 [4.1 – 27.6]	10.4 [2.1 – 18.7]
**Stimulated with aCD3**			
$CD69^+$	17.6 [2.8 – 18.7]	7.6 [1.1 – 24.9]	6.5 [0.2 – 22.6]
$IL2^+CD69^+$	2.5 [0.0 – 6.1]	1.2 [0.1 – 5.7]	1.0 [0.0 – 2.9]
$IFN\gamma^+CD69^+$	6.4 [0.0 – 17.9]	6.3 [0.0 – 29.3]	4.1 [0.0 – 10.0]
$TNF\alpha^+CD69^+$	11.5 [0.7 – 12.8]	10.0 [1.9 – 23.1]	7.7 [1.5 – 14.3]
**Stimulated with PHA**			
$CD69^+$	45.6 [21.0 – 55.6]	18.3 [9.7 – 53.6]	31.1 [5.9 – 66.4]
$IL2^+CD69^+$	10.8 [6.9 – 18.8]	10.3 [4.7 – 19.4]	10.4 [4.7 – 17.7]
$IFN\gamma^+CD69^+$	29.3 [11.7 – 54.2]	38.5 [11.6 – 57.2]	26.7 [12.0 – 38.5]
$TNF\alpha^+CD69^+$	28.6 [13.6 – 37.7]	32.3 [17.3 – 57.2]	22.7 [14.0 – 38.8]
**Stimulated with Cocktail**			
$CD69^+$	0.7 [0.0 – 1.8]	0.2 [0.0 – 1.9]	0.2 [0.0 – 2.2]
$IL2^+CD69^+$	0.0 [0.0 – 0.3]	0.0 [0.0 – 0.5]	0.0 [0.0 – 0.6]
$IFN\gamma^+CD69^+$	0.0 [0.0 – 0.5]	0.0 [0.0 – 0.6]	0.0 [0.0 – 2.0]
$TNF\alpha^+CD69^+$	0.0 [0.0 – 0.2]	0.1 [0.0 – 1.0]	0.1 [0.0 – 2.2]

**Stimulated with PPD**			
$CD69^+$	0.5 [0.0 – 1.4]	0.3 [0.0 – 2.4]	0.2 [0.0 – 1.6]
$IL2^+CD69^+$	0.0 [0.0 – 0.0]	0.0 [0.0 – 0.1]	0.0 [0.0 – 0.2]
$IFN\gamma^+CD69^+$	0.0 [0.0 – 0.4]	0.0 [0.0 – 1.0]	0.0 [0.0 – 0.3]
$TNF\alpha^+CD69^+$	0.2 [0.0 – 1.2]	0.0 [0.0 – 1.1]	0.0 [0.0 – 1.2]

Data is presented as median % [range]. aCD3: Anti-CD3. IFNγ: Inteferon gamma. IL2: Interleukin 2. PHA: Phyto haemaglutine. PPD: Purified protein derivative Tuberculosis. SEB: Staphylococcal enterotoxin B. TNFα: Tumor necrosis factor alpha.

Supplementary Table S4. Serum cytokines

Cytokine	Responders	Non-responders
BAFF/TNF (pg/mL)	1347 [985 – 1791]	1559 [420 – 2113]
IL-2R (pg/ml)	1004 [683 –2381]	1214[178 – 6568]
IFNγ (pg/ml)	2.47 [1.44 – 3.90]	2.64 [0.36 – 13.2]
CCL17/TARC (pg/ml)	600 [245 – 999]	496 [99.7 – 3228]
CXCL13 (pg/ml)	88.7 [35.7 – 233]	59.1 [25.0 – 88.1]
IL-6 (pg/ml)	3.01 [0.61 – 31.2]	6.69 [1.76 –16.3]

Data is presented as median [range]. BAFF/TNF: B-cell activating factor/Tumor necrosis factor. CCL17/TARC: C-C Motif T-lymphocyte-associated protein 4/Thymus and activation regulated chemokine. CXCL13:C-X-C Motif Chemokine Ligand 13. IFNγ: Interferon gamma. IL-2R: Interleukin 2 receptor. IL-6: Interleukin 6.
